# Morphology and Phylogeny Reveal Five Novel Species in the Genus *Cordyceps* (Cordycipitaceae, Hypocreales) From Yunnan, China

**DOI:** 10.3389/fmicb.2022.846909

**Published:** 2022-04-13

**Authors:** Quan-Ying Dong, Yao Wang, Zhi-Qin Wang, De-Xiang Tang, Zhi-Yuan Zhao, Hui-Juan Wu, Hong Yu

**Affiliations:** ^1^Yunnan Herbal Laboratory, College of Ecology and Environmental Sciences, Yunnan University, Kunming, China; ^2^The International Joint Research Center for Sustainable Utilization of Cordyceps Bioresources in China and Southeast Asia, Yunnan University, Kunming, China

**Keywords:** entomopathogenic fungi, multilocus phylogeny, new taxon, species diversity, taxonomy

## Abstract

The current study was aimed to introduce five new species of *Cordyceps* from Yunnan, with morphological descriptions, illustrations, color photographs, phylogenetic placement, associated host, and a comparison with allied taxa. The five new species were morphologically distinct from all other *Cordyceps sensu lato* species, and it was also suggested that they should differ from other species in the genus *Cordyceps* based on combined multigene analyses. Employing DNA nucleotide sequences of the nr*LSU*, nr*SSU*, *tef-1*α, *rpb1*, and *rpb2*, the five new species were recognized in the clade of *Cordyceps* by using molecular phylogenetic analyses, including five well-supported subclades: three new species, *Cordyceps bullispora, Cordyceps longiphialis*, and *Cordyceps nabanheensis*, were found in the subclade of *C. pruinosa*, and two new species, *Cordyceps pseudotenuipes* and *Cordyceps simaoensis*, were located in the subclade of *C. tenuipes*. The five novel species shared similar morphologies to other species in the genus *Cordyceps*, with fleshy and brightly pigmented stromata; perithecia superficial to completely immersed, ordinal in arrangement; and hyaline asci, with thickened cylindrical ascus apex. The morphological characteristics of 66 species in *Cordyceps sensu stricto*, namely, 5 novel species and 61 known taxa, were also compared.

## Introduction

*Cordyceps* Fr. is a well-known genus of arthropod-pathogenic fungi. It was shown that many species of *Cordyceps* played a significant role in the cycling of matter in an ecological system, had a high ecological and economic value for biocontrol and bioactive compounds, and served as a model system for research on fungal insect pathology (Zha et al., 2018; Chen W. H. et al., 2019). Complexes of some cordycipitoid fungal species and their natural host, such as *C. militaris* Fr., *C. chanhua* Z. Z. Li, F. G. Luan, Hywel-Jones, C. R. Li and S. L. Zhang, and *C. kyusyuensis* Kawam, have received significant attention in traditional medicine industry due to the detection of bioactive compounds with anti-aging, anti-tumor, antioxidant, anti-inflammatory, and immuno-modulatory effects (Castillo et al., 2018; Zhao et al., 2018; Lou et al., 2019; Li et al., 2021; Zhang et al., 2021). *Cordyceps tenuipes* (Peck) Kepler, B. Shrestha and Spatafora has been applied in a variety of functional foods in Japan and South Korea, possessing nutritional, immune-regulatory, antitumor, analgesic, antibacterial, and anti-malaria effects (Wang Y. et al., 2020). *Cordyceps* species reproduce *via* sexual (ascospores) or asexual (conidia) spores, or both (Mora et al., 2017). The host range of *Cordyceps* embraces 7 orders of Arthropoda, namely, Araneae, Coleopteran, Dermaptera, Hemiptera, Hymenoptera, Lepidoptera, and Orthoptera, where Coleoptera and Lepidoptera are the two significant orders to host beyond the estimated 200 *Cordyceps* spp. as recorded (Shrestha et al., 2016; Kepler et al., 2017; Mongkolsamrit et al., 2020; Wang Y. B. et al., 2020).

Fries (1818) was credited with coining *Cordyceps* as a genus in *Pyrenomycetes* from a hybrid of the Greek word *cordyle* and the Latin word *caput*, meaning a club and a head, respectively. However, this genus was then treated as a tribe level of *Sphaeria* and described as stroma erect, stipe simple or branching, a sterile stalk supporting the perithecia at the periphery, and projecting openings at the apex (Shrestha et al., 2014). Nearly 20 different genera have been accounted as synonyms of *Cordyceps* in various sources (Shrestha et al., 2014). Shrestha et al. (2014) provided a comprehensive review regarding the taxonomic history of *Cordyceps* and concluded that the genus is the oldest valid genus in Cordycipitaceae (Hypocreales, Ascomycota) and is typified by a sexual morph. Owing to the cylindrical shape of the stroma, *C. militaris*, the type species of *Cordyceps*, was already described in the 17th- and early 18th-century literature (Shrestha et al., 2014).

Species of Cordycipitaceae produce three types of ascospore, namely disarticulating ascospores (e.g., *C. militaris*), intact ascospores [e.g., *Blackwellomyces cardinalis* (G. H. Sung and Spatafora) Spatafora and Luangsa-ard and *Blackwellomyces pseudomilitaris* Hywel-Jones and Sivichai], and bola-ascospores (e.g., *C. bifusispora* O. E. Eriksson), with superficial to partially immersed perithecia on fleshy stromata that are pallid to brightly pigmented. Currently, *Cordyceps s. l.* (Fr) Link (1833) consists of approximately 1,300 known species assigned to three families (Cordycipitaceae, Ophiocordycipitaceae, and partial Clavicipitaceae) in the order Hypocreales (Sung et al., 2007; Zha et al., 2018). Progressive works from researchers worldwide have enriched the account of species diversity within Cordycipitaceae and revealed new genera (Kepler et al., 2017; Mongkolsamrit et al., 2018, 2020; Wang Y. B. et al., 2020). Up to now, the Cordycipitaceae has twenty genera: *Akanthomyces* Lebert, *Amphichorda* Fr., *Ascopolyporus* Möller, *Beauveria* Vuill, *Blackwellomyces* Spatafora and Luangsa-ard, *Cordyceps s. s.*, *Engyodontium* de Hoog, *Flavocillium* H. Yu, Y. B. Wang, Y. Wang, Q. Fan and Zhu L. Yang, *Gamszarea* Z. F. Zhang and L. Cai, *Gibellula* Cavara, *Hevansia* Luangsa-ard, Hywel-Jones and Spatafora, *Hyperdermium* J. F. White, R. F. Sullivan, Bills and Hywel-Jones, *Lecanicillium* W. Gams and Zare, *Leptobacillium* Zare and W. Gams, *Liangia* H. Yu, Y. B. Wang, Y. Wang, Z. H. Chen and Zhu L. Yang, *Neotorrubiella* Tasan., Thanakitp. and Luangsa-ard, *Parengyodontium* C. C. Tsang, J. F. W. Chan, W. M. Pong, J. H. K. Chen, A. H. Y. Ngan, M. Cheung, C. K. C. Lai, D. N. C. Tsang, S. K. P. Lau and P. C. Y. Woo, *Samsoniella* (G. Sm.) Mongkols, Noisrip, Thanakitp, Spatafora and Luangsa-ard, *Simplicillium* W. Gams and Zare, and *Torrubiella* W. Gams and Zare.

The unispecific genus *Phytocordyceps* possesses bola-ascospores, and because of its phylogenetic placement, it is also recognized as a member of Cordycipitaceae and thus transferred to *Cordyceps s. s*. Based on molecular phylogenetic studies or morphological descriptions, 35 names and new combinations were accepted for *Cordyceps s. s*. (Sung et al., 2007). Concerning the application of *Isaria*, in an effort to avoid the confusion with *Cordyceps*, Kepler et al. (2017) proposed the rejection of *Isaria* and combined 13 species into *Cordyceps s. s*. To date, together with the species as reported in recent years, there are about 70 species whose phylogenetic positions are known in *Cordyceps* worldwide (Tasanathai et al., 2016; Chirivi et al., 2017; Catania et al., 2018; Mongkolsamrit et al., 2018, 2020; Olatunji et al., 2018; Crous et al., 2019; Hyde et al., 2019; Li et al., 2020; Wang Y. B. et al., 2020; Hu et al., 2021).

Harvesting wild specimens have become a challenge as natural fungi populations are declining and large-scale cultivation has not been achieved. Recently, the demand for cultivable fungal species with medicinal applications has been increasing worldwide. Yunnan Province and the Tibetan plateau are home to a large diversity of entomogenous fungi (Chen Z. H. et al., 2019). *C. chaetoclavata* H. Yu, Y.B. Wang, Y. Wang, Q. Fan and Zhu L. Yang, *C. cocoonihabita* H. Yu, Y.B. Wang, Y. Wang, Q. Fan and Zhu L. Yang, *C. shuifuensis* H. Yu, Y. B. Wang, Y. Wang and Zhu L. Yang, and *C. subtenuipes* H. Yu, Y. B. Wang, Y. Wang, D. E. Duan and Zhu L. Yang have been described from this area (Wang Y. B. et al., 2020). In this study, based on macro- and micro-morphological characteristics, ecological data together with DNA nucleotide sequence analyses of the nuclear ribosomal large subunit (nr*LSU*) and small subunit (nr*SSU*), the genes encoding translation elongation factor 1-α (*tef-1*α), the largest subunit of RNA polymerase II (*rpb1*), and the second-largest subunit of RNA polymerase II (*rpb2*), the fungus’ phylogenetic position was assessed. Furthermore, we have also compared the morphological characteristics of 66 species in *Cordyceps s. s*., consisting of 5 novel species and 61 known taxa.

## Materials and Methods

### Sampling

*Cordyceps* samples were newly collected from Kunming, Weishan, Jinghong, and Pu’er of Yunnan, southwestern China. Voucher specimens were deposited in the YHH (Yunnan Herbal Herbarium) of Yunnan University. The isolated strains were deposited in YFCC (Yunnan Fungal Culture Collection) of Yunnan University.

Fungal materials, including the hosts, were photographed and recorded. Isolation of the fungi was achieved as per Wang Y. B. et al. (2020). The stromata or synnemata were cut into 5-mm-long segments, followed by surface sterilization with 30% H_2_O_2_ for 30 s to 1 min, and then rinsed with sterile water five times, dried with sterilized filter paper. Then, a part of the insect body was cut off, and the resulting segments and insect body were inoculated onto potato dextrose agar (PDA: potato 200 g/L, dextrose 20 g/L, and agar 20 g/L) plates containing 0.1 g/L streptomycin and 0.05 g/L tetracycline.

### Morphological Studies

Given the field notes, color images of the materials, and complementary literature data, macro-morphological characteristics, such as the host, fungi location, color, and shape of the stromata, and perithecial orientation (superficial, immersed, semi-immersed; ordinal or oblique) were examined under a dissecting microscope (Olympus SZ61), where the insect hosts were recognized with the support from professional entomologists.

The sexual characteristics, such as perithecia, asci, and ascospores, were firstly mounted on glass slides with lactophenol cotton blue solution after removing from the stroma, whereas the asexual characteristics, such as phialides and conidia, were firstly inoculated on glass slides with a thin layer of PDA medium block (5–10 mm in diameter) and overlaid by a cover slip, and cultivated in Petri dishes with a small amount of water under room temperature until further development of phialides and conidia. Then, the micro-morphological descriptions in both size and shape were determined with Olympus CX40 and BX53 microscopes, and FEI Quanta 200 scanning electron microscope. Twenty to thirty individual length and width measurements were taken, with absolute minima and maxima. Mycelia were inoculated on PDA plates and incubated at 25°C for a couple of days, and the colonies were photographed and measured every week.

### Molecular Studies

#### DNA Extraction and PCR Amplification

Total DNA was extracted from the fungal mycelia on PDA plates or herbarium materials using the modified CTAB procedure (Doyle and Doyle, 1987). For DNA amplification, the primer pairs nr*SSU*-CoF and nr*SSU*-CoR (Wang Y. B. et al., 2015) and LR5 and LR0R (Vilgalys and Hester, 1990; Rehner and Samuels, 1994) were used for the nr*SSU* and nr*LSU*, EF1α-EF and EF1α-ER (Bischoff et al., 2006; Sung et al., 2007) were used to amplify the translation elongation factor 1α (*tef-1*α), and primers RPB1-5’F and RPB1-5’R, and RPB2-5’F and RPB2-5’R (Bischoff et al., 2006; Sung et al., 2007) were used to amplify the largest and second-largest subunits of RNA polymerase II (*rpb1* and *rpb2*), respectively.

The polymerase chain reaction (PCR) matrix was composed of 2.5 μl of PCR 10 × Buffer (2 mmol/L Mg^2+^) (Transgen Biotech, Beijing, China), 0.25 μl of Taq DNA polymerase (Transgen Biotech, Beijing, China), 2 μl of dNTP (2.5 mmol/L), 1 μl of DNA template (500 ng/μl), 1 μl of forward primers (10 μmol/L), 1 μl of reverse primers (10 μmol/L), and 17.25 μl of sterile ddH_2_O. Amplification reactions were performed in a BIO-RAD T100™ thermal cycler (BIO-RAD Laboratories, Hercules, CA, United States). The PCR program was performed as described by Sung et al. (2007) and Wang Y. B. et al. (2020). Products of PCR were purified with the Gel Extraction and PCR Purification Combo Kit Beijing Genomics Institute (Shenzhen, China) and then sequenced on an automatic sequence analyzer (BGI Co., Ltd, Shenzhen, China) using the same primers as those used in amplification.

#### DNA Sequence Alignments

The samples’ nr*SSU*, nr*LSU*, *tef-1*α, *rpb1*, and *rpb2* nucleotide sequences were compared with those deposited in the GenBank database. To understand the relationship of our sample with those in the GenBank, nr*SSU*, nr*LSU*, *tef-1*α, *rpb1*, and *rpb2* sequences of the representative *Cordyceps s. s.* species available in GenBank were retrieved and combined with our sequences (Table 1). Five datasets, the nr*SSU*, nr*LSU*, *tef-1*α, *rpb1*, and *rpb2* sequences, were aligned and manually checked on Bioedit v7.0.9 (Hall, 1999). In order to examine phylogenetic conflicts among these datasets, the partition homogeneity (PH) test was performed with 1,000 randomized replicates, using heuristic searches with simple addition of sequences in PAUP* 4.0b10 (Swofford, 2002); the phylogenetic signals in the five gene markers showed no conflict.

**TABLE 1:** Names, voucher information, host, and corresponding GenBank accession numbers of the taxa used in this study.

Taxon	Voucher information	Host	GenBank Accession Number					References
			nr*SSU*	nr*LSU*	*tef-1*α	*rpb1*	*rpb2*	
*Cordyceps albocitrina*	spat 07-174		MF416575		MF416467	MF416629		Kepler et al., 2017
*Cordyceps amoene-rosea*	CBS 107.73	Coleoptera (pupa)	AY526464	MF416550	MF416494	MF416651	MF416445	Luangsa-ard et al., 2005
*Cordyceps amoene-rosea*	CBS 729.73	Coleoptera; Nitidulidae	MF416604	MF416551	MF416495	MF416652	MF416446	Luangsa-ard et al., 2005
*Cordyceps araneae*	BCC 85065	Arachnid; Araneae		MT003037	MT017850	MT017810	MT017828	Mongkolsamrit et al., 2020
*Cordyceps araneae*	BCC 85066	Arachnid; Araneae		MT003038	MT017851	MT017811	MT017829	Mongkolsamrit et al., 2020
*Cordyceps araneae*	BCC 88291	Arachnid; Araneae		MT003039	MT017852	MT017812	MT017830	Mongkolsamrit et al., 2020
*Cordyceps bifusispora*	spat 08-129		MF416576	MF416523	MF416468	MF416630		Kepler et al., 2017
*Cordyceps bifusispora*	spat 08-133.1		MF416577	MF416524	MF416469	MF416631	MF416434	Kepler et al., 2017
*Cordyceps bifusispora*	EFCC 5690	Lepidopteran (pupa)	EF468952	EF468806	EF468746	EF468854	EF468909	Sung et al., 2007
*Cordyceps bifusispora*	EFCC 8260	Lepidopteran (pupa)	EF468953	EF468807	EF468747	EF468855	EF468910	Sung et al., 2007
*Cordyceps blackwelliae*	TBRC 7255	Coleoptera (larva)		MF140703	MF140823	MF140772	MF140796	Mongkolsamrit et al., 2018
*Cordyceps blackwelliae*	TBRC 7256	Coleoptera (larva)		MF140702	MF140822	MF140771	MF140795	Mongkolsamrit et al., 2018
*Cordyceps blackwelliae*	YFCC 856	Lepidoptera (larva)	MW181780	MW173992	MW168233	MW168199	MW168216	Unpublished
*Cordyceps brevistroma*	BCC 78209	Lepidoptera (larva)		MT003044	MT017855	MT017817	MT017835	Mongkolsamrit et al., 2020
*Cordyceps brevistroma*	BCC 79253	Lepidoptera (larva)		MT003045	MT017856		MT017836	Mongkolsamrit et al., 2020
***Cordyceps bullispora***	**YFCC 8400**	**Lepidopteran (pupa)**	**OL468555**	**OL468575**	**OL473523**	**OL739569**	**OL473534**	**This study**
***Cordyceps bullispora***	**YFCC 8401**	**Lepidopteran (pupa)**	**OL468556**	**OL468576**	**OL473524**	**OL739570**	**OL473535**	**This study**
*Cordyceps caloceroides*	MCA 2249	Araneae	MF416578	MF416525	MF416470	MF416632		Kepler et al., 2017
*Cordyceps cateniannulata*	CBS 152.83	Coleoptera (adult)	AY526465	MG665226	JQ425687			Luangsa-ard et al., 2004; Mongkolsamrit et al., 2018
*Cordyceps cateniobliqua*	YFCC 3367	Coleopteran adult	MN576765	MN576821	MN576991	MN576881	MN576935	Wang Y. B. et al., 2020
*Cordyceps cateniobliqua*	YFCC 5935		MN576766	MN576822	MN576992	MN576882	MN576936	Wang Y. B. et al., 2020
*Cordyceps cateniobliqua*	CBS 153.83	Adoxophyes privatana	AY526466		JQ425688		MG665236	Luangsa-ard et al., 2004; Mongkolsamrit et al., 2018
*Cordyceps* cf. *ochraceostromata*	ARSEF 5691		EF468964	EF468819	EF468759	EF468867	EF468921	Kepler et al., 2012
*Cordyceps cf. pruinosa*	spat 08-115		MF416586	MF416532	MF416476	MF416635	MF416439	Kepler et al., 2017
*Cordyceps cf. pruinosa*	spat 09-021		MF416587	MF416533	MF416477	MF416636		Kepler et al., 2017
*Cordyceps* cf. *pruinosa*	NHJ 10627	Limacodid pupa (Lepidoptera)	EF468967	EF468822	EF468763	EF468870		Sung et al., 2007
*Cordyceps* cf. *pruinosa*	NHJ 10684	Limacodid pupa (Lepidoptera)	EF468968	EF468823	EF468761	EF468871		Sung et al., 2007
*Cordyceps* cf. *pruinosa*	EFCC 5693		EF468966	EF468821	EF468762	EF468869		Sung et al., 2007
*Cordyceps* cf. *pruinosa*	EFCC 5197		EF468965	EF468820	EF468760	EF468868		Sung et al., 2007
*Cordyceps* cf. *takaomontana*	NHJ 12623	Lepidoptera	EF468984	EF468838	EF468778	EF468884	EF468932	Sung et al., 2007
*Cordyceps chaetoclavata*	YHH 15101		MN576722	MN576778	MN576948	MN576838	MN576894	Wang Y. B. et al., 2020
*Cordyceps chiangdaoensis*	BCC 68469	Coleoptera		MF140732	KT261403			Tasanathai et al., 2016; Mongkolsamrit et al., 2018
*Cordyceps chiangdaoensis*	YFCC 857	Coleoptera: Elateridae	MW181781	MW173993	MW168234	MW168200	MW168217	
*Cordyceps cicadae*	GACP 07071701	Hemiptera	MK761207	MK761212	MK770631			Zha et al., 2019
*Cordyceps cicadae*	RCEF HP090724-31	Hemiptera: Cicadidae	MF416605	MF416552	MF416496	MF416653	MF416447	Kepler et al., 2017
*Cordyceps cocoonihabita*	YFCC 3415		MN576723	MN576779	MN576949	MN576839	MN576895	Wang Y. B. et al., 2020
*Cordyceps cocoonihabita*	YFCC 3416		MN576724	MN576780	MN576950	MN576840	MN576896	Wang Y. B. et al., 2020
*Cordyceps coleopterorum*	CBS 110.73	Coleoptera (larva)	JF415965	JF415988	JF416028	JN049903	JF416006	Kepler et al., 2012
*Cordyceps exasperata*	MCA 2288	Lepidoptera (larva)	MF416592	MF416538	MF416482	MF416639		Kepler et al., 2017
*Cordyceps farinosa*	CBS 111113		AY526474	MF416554	MF416499	MF416656	MF416450	Luangsa-ard et al., 2004; Kepler et al., 2017
*Cordyceps fumosorosea*	YFCC 4561	Lepidoptera	MN576761	MN576817	MN576987	MN576877	MN576931	Wang Y. B. et al., 2020
*Cordyceps fumosorosea*	CBS 244.31	Butter	MF416609	MF416557	MF416503	MF416660	MF416454	Kepler et al., 2017
*Cordyceps fumosorosea*	CBS 375.70	Food			MF416501	MF416658	MF416452	Kepler et al., 2017
*Cordyceps fumosorosea*	CBS 107.10			MG665227	HM161735		MG665237	Luangsa-ard et al., 2005
*Cordyceps grylli*	MFLU 17-1023		MK863048	MK863055	MK860193			Unpublished
*Cordyceps grylli*	MFLU 17-1024		MK863049	MK863056	MK860194			Unpublished
*Cordyceps inthanonensis*	BCC 79828	Lepidoptera (pupa)			MT017854	MT017816	MT017833	Mongkolsamrit et al., 2020
*Cordyceps inthanonensis*	BCC 56302	Lepidoptera (pupa)		MT003040	MT017853	MT017814	MT017831	Mongkolsamrit et al., 2020
*Cordyceps inthanonensis*	BCC 55812	Lepidoptera (larva)		MT003041	–	MT017815	MT017832	Mongkolsamrit et al., 2020
*Cordyceps jakajanicola*	BCC 79816	Hemiptera		MN275696	MN338479	MN338484	MN338489	Crous et al., 2019
*Cordyceps jakajanicola*	BCC 79817	Hemiptera		MN275697	MN338480	MN338485	MN338490	Crous et al., 2019
*Cordyceps javanica*	TBRC 7259	Lepidoptera		MF140711	MF140831	MF140780	MF140804	Mongkolsamrit et al., 2018
*Cordyceps javanica*	CBS 134.22	Coleoptera	MF416610	MF416558	MF416504	MF416661	MF416455	Kepler et al., 2017
*Cordyceps kuiburiensis*	BCC 90322	Araneidae		MK968816	MK988032	MK988030		Crous et al., 2019
*Cordyceps kuiburiensis*	BCC 90323	Araneidae		MK968817	MK988033	MK988031		Crous et al., 2019
*Cordyceps kyusyuensis*	EFCC 5886	Lepidoptera (pupa)	EF468960	EF468813	EF468754	EF468863	EF468917	Sung et al., 2007
*Cordyceps lepidopterorum*	TBRC 7263	Lepidoptera (larva)		MF140699	MF140819	MF140768	MF140792	Mongkolsamrit et al., 2018
*Cordyceps lepidopterorum*	TBRC 7264	Lepidoptera (larva)		MF140700	MF140820	MF140769	MF140793	Mongkolsamrit et al., 2018
***Cordyceps longiphialis***	**YFCC 8402**	**Rotten wood**	**OL468557**	**OL468577**	**OL473525**	**OL739571**	**OL473536**	**This study**
***Cordyceps longiphialis***	**YFCC 8403**	**Rotten wood**	**OL468558**	**OL468578**	**OL473526**	**OL739572**	**OL473537**	**This study**
*Cordyceps militaris*	YFCC 6587	Lepidoptera (pupa)	MN576762	MN576818	MN576988	MN576878	MN576932	Wang Y. B. et al., 2020
*Cordyceps militaris*	YFCC 5840	Lepidoptera (pupa)	MN576763	MN576819	MN576989	MN576879	MN576933	Wang Y. B. et al., 2020
*Cordyceps morakotii*	BCC 55820	Hymenoptera (ant pupa)		MF140730	KT261399			Tasanathai et al., 2016
*Cordyceps morakotii*	BCC 68398	Hymenoptera (ant pupa)		MF140731	KT261398			Tasanathai et al., 2016
***Cordyceps nabanheensis***	**YFCC 8409**	**Lepidopteran**	**OL468564**	**OL468584**	**OL473532**	**OL739578**	**OL473543**	**This study**
***Cordyceps nabanheensis***	**YFCC 8410**	**Lepidopteran**	**OL468565**	**OL468585**	**OL473533**	**OL739579**	**OL473544**	**This study**
*Cordyceps neopruinosa*	BCC 91361	Lepidoptera (pupa)		MT003047	MT017858		MT017838	Mongkolsamrit et al., 2020
*Cordyceps neopruinosa*	BCC 91362	Lepidoptera (pupa)		MT003048	MT017859	MT017818	MT017839	Mongkolsamrit et al., 2020
*Cordyceps nidus*	HUA 186125	Araneae (Mygalomorphae)	KC610778	KC610752	KC610722		KC610711	Chirivi et al., 2017
*Cordyceps nidus*	HUA 186186	Araneae (Mygalomorphae)	KY360301	KC610753	KC610723	KY360297		Chirivi et al., 2017
*Cordyceps ninchukispora*	EGS 38.165	Plant (*Beilschmiedia erythrophloia*)	EF468991	EF468846	EF468795	EF468900		Sung et al., 2007
*Cordyceps ninchukispora*	EGS 38.166	Plant (*Beilschmiedia erythrophloia*)	EF468992	EF468847	EF468794	EF468901		Sung et al., 2007
*Cordyceps ningxiaensis*	HMJAU 25074			KF309671				Yan and Bau, 2015
*Cordyceps ningxiaensis*	HMJAU 25076			KF309673				Yan and Bau, 2015
***Cordyceps pseudotenuipes***	**YFCC 8404**	**Lepidoptera**	**OL468559**	**OL468579**	**OL473527**	**OL739573**	**OL473538**	**This study**
***Cordyceps pseudotenuipes***	**YFCC 8405**	**Lepidoptera**	**OL468560**	**OL468580**	**OL473528**	**OL739574**	**OL473539**	**This study**
*Cordyceps oncoperae*	ARSEF 4358	Lepidoptera; Oncopera intricate	AF339581	AF339532	EF468785	EF468891	EF468936	Sung et al., 2007
*Cordyceps polyarthra*	MCA 996		MF416597	MF416543	MF416487	MF416644		Kepler et al., 2017
*Cordyceps polyarthra*	MCA 1009	Lepidoptera	MF416598	MF416544	MF416488	MF416645		Kepler et al., 2017
*Cordyceps pruinosa*	ARSEF 5413	Lepidoptera: Limacodidae	AY184979	AY184968	DQ522351	DQ522397	DQ522451	Spatafora et al., 2007
*Cordyceps qingchengensis*	MFLU 17-1022	Lepidoptera; Bombycidae	MK761206	MK761211	MK770630			Zha et al., 2019
*Cordyceps rosea*	spat 09-053	Lepidopteran larva	MF416590	MF416536	MF416480	MF416637	MF416442	Kepler et al., 2017
*Cordyceps roseostromata*	ARSEF 4871		AF339573	AF339523				Sung et al., 2001
*Cordyceps shuifuensis*	YFCC 5230		MN576721	MN576777	MN576947	MN576837	MN576893	Wang Y. B. et al., 2020
***Cordyceps simaoensis***	**YFCC 8406**	**Lepidoptera**	**OL468561**	**OL468581**	**OL473529**	**OL739575**	**OL473540**	**This study**
***Cordyceps simaoensis***	**YFCC 8407**	**Lepidoptera**	**OL468562**	**OL468582**	**OL473530**	**OL739576**	**OL473541**	**This study**
***Cordyceps simaoensis***	**YFCC 8408**	**Lepidoptera**	**OL468563**	**OL468583**	**OL473531**	**OL739577**	**OL473542**	**This study**
*Cordyceps* sp.	CBS 102184	Spider (Arachnida)	AF339613	AF339564	EF468803	EF468907	EF468948	Kepler et al., 2012
*Cordyceps* sp.	EFCC 2535		EF468980	EF468835	EF468772			Sung et al., 2007
*Cordyceps* sp.	YFCC 5833		MN576764	MN576820	MN576990	MN576880	MN576934	Wang Y. B. et al., 2020
*Cordyceps spegazzinii*	ARSF 7850			DQ196435				Torres et al., 2005
*Cordyceps subtenuipes*	YFCC 6051		MN576719	MN576775	MN576945	MN576835	MN576891	Wang Y. B. et al., 2020
*Cordyceps subtenuipes*	YFCC 6084		MN576720	MN576776	MN576946	MN576836	MN576892	Wang Y. B. et al., 2020
*Cordyceps succavus*	MFLU 18-1890		MK086058	MK086062		MK084616	MK079353	Unpublished
*Cordyceps tenuipes*	TBRC 7265	Lepidopteran (pupa)		MF140707	MF140827	MF140776	MF140800	Mongkolsamrit et al., 2018
*Cordyceps tenuipes*	TBRC 7266	Lepidopteran (pupa)		MF140708	MF140828	MF140777	MF140801	Mongkolsamrit et al., 2018
*Cordyceps tenuipes*	ARSEF 5135	Lepidopteran (pupa)	MF416612	JF415980	JF416020	JN049896	JF416000	Kepler et al., 2012, 2017
*Cordyceps tenuipes*	YFCC 4266		MN576774	MN576830	MN577000	MN576890	MN576944	Wang Y. B. et al., 2020
*Cordyceps yinjiangensis*	YJ 06221	Ant			MT577003		MT577002	Li et al., 2020
*Liangia sinensis*	YFCC 3103		MN576726	MN576782	MN576952	MN576842	MN576898	Wang Y. B. et al., 2020
*Liangia sinensis*	YFCC 3104		MN576727	MN576783	MN576953	MN576843	MN576899	Wang Y. B. et al., 2020

*Boldface: data generated in this study.*

#### Phylogenetic Analyses

Phylogenetic trees were visualized with FigTree v1.4.0 (Rambaut, 2006) and edited in Microsoft PowerPoint, then saved as .PDF format and finally converted to .JPG format using Adobe Illustrator CS6 (Adobe Systems Inc., United States). The finalized alignments and trees were submitted in TreeBASE (Submission ID: 29339).

Phylogenetic analyses of the concatenated five-gene datasets were conducted using ML and BI methods. The GTR + I + G were chosen as the best models for nr*SSU*-nr*LSU-tef-1α-rpb1-rpb2*, using the Akaike Information Criterion (AIC) implemented in MrModeltest v 2.3 (Nylander, 2004), and then the partitioned analyses were separately conducted. For ML analyses, raxml v 8.2.7 was employed. All parameters were kept as default with an exception that the model was chosen as GTRGAMMAI. The statistic supports were calculated using 1,000 replicates of non-parametric bootstrapping. BI analysis was carried out with MrBayes v 3.2.6 using the selected models for 5 million generations with the value of *stopval* set to 0.01 *via* the *stoprul* command. At the same time, other parameters were kept as default and trees were summarized. Statistic supports were obtained using *sumt* command complemented in MrBayes by discarding the first 25% generations as burn-ins. The Bayesian trees were sampled every 100 generations. The first 25% trees were discarded as burn-ins, and the remaining trees were employed to create a consensus tree using *sumt* command.

## Results

### Sequence Alignment

The combined 101-taxon 5-gene dataset consisted of 4,627 base pairs of sequence data (nr*SSU* 1060 bp, nr*LSU* 877 bp, *tef-1*α 999 bp, *rpb1* 719 bp, and *rpb2* 972 bp). A total of 1,039 were parsimony-informative (nr*SSU* 45 bp, nr*LSU* 77 bp, *tef-1*α 370 bp, *rpb1* 234 bp, and *rpb2* 313 bp). A total of 101 taxa were complete for all five genes, and the number of taxa for each gene was as follows: nr*SSU* 71 taxa, nr*LSU* 97 taxa, *tef-1*α 95 taxa, *rpb1* 82 taxa, and *rpb2* 72 taxa (Table 1).

### Molecular Phylogeny

In this study, we generated nr*SSU*, nr*LSU*, *tef-1*α, *rpb1*, and *rpb2* sequences by ten living cultures and one wild material, and their accession numbers are shown in Table 1. Sequences of *Liangia sinensis* YFCC 3103 and YFCC 3104 in the *Cordycipitaceae* were chosen as outgroups in the phylogenetic analyses.

**FIGURE 1:**
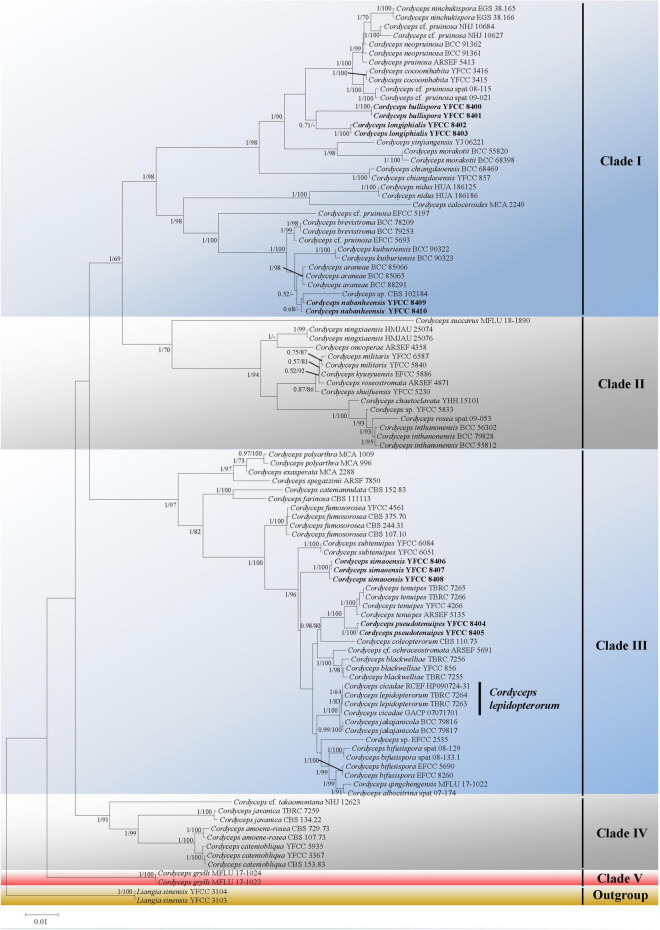
Both ML and BI analyses generate a phylogenetic tree from the concatenated nr*SSU*, nr*LSU*, *tef-1*α, *rpb1*, and *rpb2* datasets. There were no discrepancies between the topology resulting from Bayesian and ML analysis for supported nodes. Bootstrap values (≥60%) derived from ML analyses and posterior probabilities from Bayesian inference (≥0.50) are shown either above or beneath the branches of the nodes. Isolates in bold type are those analyzed in this study.

Five major (I–IV) clades and five new species could be recognized in *Cordyceps s. s.* (Figure 1); collections from southwestern China were grouped into five separate species (in boldface, see below) (*C. bullispora, C. longiphialis*, and *C. nabanheensis* in clade I, and *C. pseudotenuipes* and *C. simaoensis* in clade III). Clade I included *C. pruinose* and 19 other species, with 98% bootstrap support and 1 Bayesian PP support (Figure 1). In clade II, *C. militaris* and eight other species were grouped together (BS = 70%, PP = 1) (Figure 1). Clade III harbored *C. tenuipes* and 18 other taxa (BS = 97%, PP = 1) (Figure 1). Clade IV included *C.* cf. *takaomontana* NHJ 12623, *C. javanica, C. amoenerosea*, and *C. cateniobliqua* (BS = 91%, PP = 1) (Figure 1). Clade V included only two exemplars of the species, *C. grylli* (Figure 1).

## Taxonomy

The key morphological characteristics that distinguish current *Cordyceps s. s.* species are summarized in the literature (Tables 2, 3). Including the five new species, there are 66 species of *Cordyceps s. s.* involved in the current study, among which we have compared 51 species of the sexual morphs in *Cordyceps s. s.* in Table 2 and 38 species of the asexual morphs in *Cordyceps s. s.* in Table 3.

**TABLE 2:** Comparison between the sexual morphs in *Cordyceps.*

Species	Stromata (mm)	Fertile part (mm)	Perithecia (μm)	Asci (μm)	Ascospores (μm)	Part-spores (μm)	References
*Cordyceps araneae*	Solitary or gregarious, 4–8 × 0.5	Clavate, elliptical to fusiform, 2.5– 5 × 1–2	Semi-immersed, 450–500 × 150–200	Cylindrical, 8-spored, (60)145–220(250) × 2–2.5	Bola-shaped, (250)280–340(400), central part filiform, 0.3 μm broad, 3 or 4-septa		Mongkolsamrit et al., 2020
*Cordyceps belizensis*	100	20 × 9	Immersed, 480–570 × 260–300	Cylindrical, 260–300 × 6	Filiform	4–8 × 1.5	Mains, 1940
*Cordyceps bifusispora*	Simple, 13 × 0.5–0.7	Cylindrical, 6 × 1.3	Immersed, 300 × 150–170	Cylindrical, 8-spored, 200–300 × 3–4.5	Bifusiform, 3-septa, 145–220 × 0.4–1.6		Eriksson, 1982
*Cordyceps blackwelliae*	Gregarious, cylindrical to clavate, 8–10 × 1–1.5	4–6 × 1.5–2	Superficial, (300–)302–332 (–350) × (150–)155–189 (–200)	Cylindrical, 8-spored, 300 × 2.5–3	Bola-shaped, 3- or 4-septa, (250–)259–283.5 (–290) × 1		Mongkolsamrit et al., 2018
*Cordyceps brasiliensis*	Sub-solitary or branch, 60–73 × 1.5–2	15–20 × 3–5	Immersed, 350–700 × 175–315	Worm-form, 70–333 × 3.5–5	Filiform	3.5–7 × 0.7	Fang et al., 1995
*Cordyceps brevistroma*	Solitary or gregarious, 2.5–10 × 0.5	Clavate, subglobose, 2–5 × 1–1.5	Semi-immersed, ovoid, 250–330(350) × 130–185(200)	Cylindrical, 8-spored, (110)140–220(250) × 2– 5	Whole, bola-shaped, 150– 200 × 0.5		Mongkolsamrit et al., 2020
***Cordyceps bullispora***	**Solitary, 10–20, rhizoids flexuous**	**Clavate, 3.5–7.4 × 0.5–1.5**					**This study**
*Cordyceps chaetoclavata*	Solitary, 23 × 0.8	Clavate, 5.6 × 0.7–1.1	Superficial, 402–610 × 280–427	Cylindrical, 8-spored, 274–385 × 3.7–4.8	Filiform, 127–260 × 0.9–1.2	Cylindrical, 3–6 μm long	Wang Y. B. et al., 2020
*Cordyceps chanhua*	Solitary, simple		Partly immersed, 475–602 × 222–319	Cylindrical, 8-spored, 235–380 × 2.1–3	Filiform, 246–360 × 1.5–1.8	6.4–13.8 × 2.1–3.1	Li et al., 2021
*Cordyceps chiangdaoensis*	Solitary to gregarious, simple or compound	2–7 × 1–1.5	Superficial, 200–450 × 70–170	Cylindrical, 8-spored, 175–315 × 2–3	Bola-shaped, 3-septa, 200–300 × 1		Tasanathai et al., 2016
*Cordyceps chichibuënsis*	Solitary, 13–15 × 1–1.5		Semi-immersed, 400 × 230–260	5.5 μm wide		1.5–2 × 1.5	Kobayasi and Shimizu, 1980
*Cordyceps chishuiensis*	Solitary or 2–3, 10–20 × 1–2	Elliptical, 5–6 × 3–4	Superficial, 340–440 × 200–240	Clavate, 165 × 6		Cylindrical, 3.6–5.4 × 1.2	Liang et al., 2002; Zha et al., 2021
*Cordyceps coccinea*	Gregarious, 4–35 × 0.3–0.5	Cylindrical or clavate, 3–4 × 1.25–1.75	Immersed, 350 × 100			Cylindrical, 2–4 × 1	Petch, 1924; Shrestha, 2011
*Cordyceps cocoonihabita*	Two or gregarious, 15.2–57.8	Clavate, 3.5–17.4 × 0.3–1.5	Superficial, 346–435 × 125–199	Cylindrical, 8-spored, 205–330 × 2.1–3.3	Cylindrical, septate, 140–269 × 1.4–2.1	Cylindrical, 2.9–8.0 μm long	Wang Y. B. et al., 2020
*Cordyceps cuncunae*	Solitary, rarely two, 59–92–105 × 7–8–10	Ovalis to subglobose, 15–18–21 × 12–15–18	Immersed, 772–793–829 × 257–279–314	Cylindrical, poriferous, 8-spored, 364–391–422 × 6–7–8	Filiform, 3–septate, 340–375–414 × 1.5–2	Cylindrical to subfusiform, 4.3–6.3–8.6	Palfner et al., 2012
*Cordyceps cylindrica*	Single, 36 × 2–2.6	Cylindrical, clavate with obtuse top, 13 × 3.7–4	Immersed, fusiform to elliptical or flask-shaped, 850–1000 × 200–225	4.5–5.5 μm wide		Truncate ends, 3–4 × 1.2	Kobayasi and Shimizu, 1977
*Cordyceps dermapteoigena*	Solitary, simple, 15 × 1	Cylindrical, 7 × 1.5, sterile apex 5 mm long	Embedded, 405–450 × 180–230	6–7.2 μm wide	Filiform, multi-septate, (4.8–)6–15 × 2–3		Liang et al., 2003
*Cordyceps doiana*	Solitary, simple, 30 × 0.3–0.4	Cylindrical, 9 × 0.7–0.8	Semisuperficial, 2/3 embedded, 250 × 170	125–135 × 6–7	4–5 × 1		Kobayasi, 1981
*Cordyceps formosana*	Cylindrical or branched, 8.7–21.6	5.3–8.0 × 1.5–4.0	Semi-immersed, 360–520 × 230–330	Linear, 230–335 × 6.0–7.2		5.0–9.8 × 1.4–2.0	Olatunji et al., 2018
*Cordyceps inthanonensis*	Multiple, 6–25 mm long	Cylindrical to clavate, half of stroma, 3–5 mm wide	Semi-immersed, ovoid, 600–720 × 220–420	Cylindrical, 450–600 × 4–6		Cylindrical, 3–4 × 1–1.5	Mongkolsamrit et al., 2020
*Cordyceps ishikariensis*	Gregarious, 45–65 × 1.5–2	Fertile part is slightly wider than stipe	Semi-immersed, 500–570 × 240–300	250–360 × 4			Shrestha, 2011
*Cordyceps jakajanicola*	Gregarious, simple, 32–45	On the terminal end c. 1/3 of the stroma	Semi-immersed, 400–650 × 300–400	Cylindrical, 265–360 × 4–5	Bola-shaped, 250–310 × 1		Crous et al., 2019
*Cordyceps kuiburienis*	Solitary, 8 × 1–1.5, rhizoids flexuous	Cavate to subglobose, 1.5–5 × 1–2.5	Pseudo-immersed, obpyriform, (350–)370–460(–550) × (120–) × 140–190(–240)	Cylindrical, 280 × 3–5	Filiform, 250 × 1		Crous et al., 2019
*Cordyceps kyusyuensis*	Multiple, 15–20 mm long	Cylindrical, 10–12 mm long	Semi-superficial, ovoid, 410–580 × 210–330	4 μm wide		4–5 × 1	Kobayasi, 1981
***Cordyceps longiphialis***	**Two, 13–25**	**Clavate, 4–15 × 2.0–2.5**	**Superficial, 380–612 × 167.5–268.3**	**Fusiform to cylindrical. 8-spored, 113–200 × 1.1–2.7**	**Bola-shaped, 110–184 × 0.8–1.3**		**This study**
*Cordyceps militaris*	Solitary or gregarious, 8–70 mm long	Clavate, half of stroma	Immersed to semi-immersed, 500–720 × 300–480	300–510 × 3.5–5	Filiform, multi-septate	2–4.5 × 1–1.5	Mains, 1958; Liang et al., 2007
*Cordyceps morakotii*	Simple or compound, 3–7 × 0.5–1	Cylindrical to obovoid	Superficial, 200–300 × 70–120	Cylindrical, 8-spored, 150–200 × 3–5	Bola-shaped, 200–250 × 1, 3-septate		Tasanathai et al., 2016
***Cordyceps nabanheensis***	**Solitary or gregarious, 14–23, rhizoids flexuous,**	**Clavate, 2.0–6.5 × 0.7–1.5**	**Superficial, 224–322.6 × 71.2–317.3**				**This study**
*Cordyceps nidus*	Gregarious, simple, 10–42 × 0.5–2	Subcylindrical, 2.5–18 × 0.5–3	Pseudoimmersed, 300–500 (–630) × 110–190 (–205)	Cylindrical, (145–) 190–360 × 2–4	Filiform, 100–120 × 1.0	(4–)6–10 × 1	Chirivi et al., 2017
*Cordyceps ninchukispora*	1–6 branches, 13.8–22.4 × 0.3–0.9	5.7–14.2 × 0.8–0.9	Superficial, 95–145 × 50–60	Long cylindrical, 75–105 × 2.1–3.1	Ninchukiform, 90–110 × 1.2, 3–4 septate		Su and Wang, 1986
*Cordyceps ningxiaensis*	1–2 branches, 5–15 × 0.3–1.2	Spherical to ovoid, 1.2–3 × 1.2–2.8	Immersed, 288–400 × 103–240	Cylindrical, 8-spored, 168–205 × (3.7–)4.1–5.5(–6.6)	Filiform, multi-septate	3.6–7.8 × 1.0–1.4	Yan and Bau, 2015
*Cordyceps oncopera*e	1–4 branches, 35 mm long,	Acute apex, 4–10 × 2–3	Ovoid, 350–410 × 180–230(–380)	Cylindrical, 8-spored, (168–)200–224 (–256) × (5–)6–6.5	Filiform, 104–139 × 1.5–2		Wright, 1993
*Cordyceps parvula*			Superficial, 500–650 × 250–300	Cylindrical, 400–500 × 5–6	Filiform	8–10 × 1	Mains, 1959
*Cordyceps parvistroma*	Solitary, 5 mm long	Subglobose, clavate, 4–10 × 2–3	Superficial, ovoid, (320)330–390(410) × (175)180–270(350)	Cylindrical, (150)175–260(300) × 2–2.5	Bola-shaped, (180)210–225(250) × 0.5		Mongkolsamrit et al., 2020
*Cordyceps polyarthra*	Gregarious, 30–44	Cylindrical to narrowly clavate, 12–14	Semi-immersed, 220–300 × 180–200	Cylindrical, 167–217.5 × 3.5–4.5	Filiform, multi-septate	Cylindrical, 4.5–11 × 0.5–1.0	Catania et al., 2018
*Cordyceps polystromata*	Gregarious, 11–37 × 3.0–9.8	Cylindrical to clavate, 5–17 × 2.3–8.6	Superficial, 522.3–663.4 × 296.4–576.7 (583.5 × 412.2)	Cylindrical, 34.2–172.8 × 4.1–6.5 (68.4 × 5.2)	Linear, 34.2–172.8 × 0.9–2.6 (68.4 × 1.7)	Cylindrical, 1.3–3.0 × 1.1–2.2 (2.1 × 1.6)	Duan, 2019
*Cordyceps pruinosa*	Solitary, 15–47.5	Narrow-clavate or subcylindrical, 7 × 1.5	Superficial, narrow-oval or ovoid-cylindrical, 400 × 100	185–200 × 2	Bifusiform and filiform, 4–6 μm wide	Cylindrical, 6 × 1	Petch, 1924; Liang, 1983; Liang et al., 2007
*Cordyceps pseudomilitaris*	1-3, simple, 12–25 × 1–2	2.5–9(–10) × 1.5–2.5	Semi-immersed, 320–500 × 225–350	Cylindrical, 210–395 × 5–6	Filiform, multi-septate, 200–380 × 1		Catania et al., 2018; Olatunji et al., 2018
*Cordyceps qingchengensis*	1-3 branches, 25 mm long	7–9 × 2.0–2.5	Partially immersed, sharply pointed, 335–490 × 145–240	Cylindrical, 8-spored, 180–200 × 2.4–4	Filiform, partly bifusiform, 180–220 × 0.45–0.65		Zha et al., 2019
*Cordyceps racemosa*	Gregarious, branched	Lanceolate, not much differentiated from the stipe					Zang and Kinjo, 1998
*Cordyceps rosea*	Solitary, 11 mm long	Cavate	Immersed, ovoid, 330–380 × 160–230	100 × 3–4	Whole, multi-septate		Kobayasi and Shimizu, 1982
*Cordyceps rostrata*	Solitary, simple, 35 mm long	Cylindrical, 10 × 2	Superficial, subglobose, 420–525 × 255–375	Cylindrical, 6 μm wide		Cylindrical, (3.6–) 4.8–6 (–7.2) × (1.2–)1.5–2	Liang et al., 2003
***Cordyceps simaoensis***	**Solitary or gregarious, 7–25.1**	**Elliptical to fusiform, 1–4.2 × 1.5–3**	**Immersed, 638.4–757.6 × 371–531.1**	**Clavate to nearly cylindrical, 8-spored, 66.9–126.1 × 1.9–2.7**			**This study**
*Cordyceps singeri*	1-2, simple, 10–20 × 0.5–1.5	Cylindrical to clavate, 1 × 3–4	Embedded, 325–520 × 220–475	Cylindrical, (187–)425–475 × 3–4(–4.5)	Filiform, multi-septate	3.0–4.0 × 1.0	Catania et al., 2018
*Cordyceps spegazzinii*	Solitary, simple, 7–9 mm long	Cylindrical to clavate, 1 × 3–4	Superficial to partially immersed, 400–460 × 200–240	Cylindrical, 8-spored, 200–250 × 2.5–3	Filiform, multi-septate, 100–250 × 0.5–1		Torres et al., 2005
*Cordyceps submilitaris*	20–30 × 1–1.5	10–25 × 2	Embedded	Cylindrical, 300–420 × 3–4	Filiform	2.0–4.0 × 0.5	Mains, 1940
*Cordyceps shuifuensis*	Solitary, 25 mm long	Clavate,4 × 1.5	Pseudoimmersed, 450–620 × 300–430	Cylindrical, 275–510 × 3.5–5.2	Filiform, multiseptate, 180–410 × 1.2–1.7	Cylindrical, 2.8–6.5 μm long	Wang Y. B. et al., 2020
*Cordyceps succavus*	Solitary, 40–50 × 3–6	Cylindrical, 15–20 × 4–5	Semi-immersed, 534–655 × 179–278	Cylindrical, 8-spored, 486–600 × 3.6–4.9	Filiform, 466–594 × 0.9–1.2	Cylindrical, 2.8–4.9 × 0.9–1.2	Hyde et al., 2019
*Cordyceps suoluoensis*	Gregarious (2–4), 15 × 2–3	Cylindrical, 7 × 3	Pseudoimmersed, 400–500 × 260–300	3.6–4.8 μm wide	Filiform	Cylindrical,(6–)9–12 × 1.8–2.0	Liang et al., 2002
*Cordyceps takaomontana*	Solitary or gregarious	Cylindrical, 8–10 × 1.5–2 mm	Superficial, 375–450 × 145–195	Filiform, 1200 × 2.4–3	Filiform	Cylindrical, 6–8 × 0.5–0.8	Liang et al., 2003, 2007
*Cordyceps translucens*	10 × 1	Globose or ovoid, 2.5 × 2	Superficial, 0.5 × 0.3	Cylindrical, 8-spored		Cylindrical, 6 × 1	Petch, 1924

*Boldface: data generated in this study.*

**TABLE 3:** Comparison between the asexual morphs in *Cordyceps.*

Species	Conidiophores (μm)	Phialides	Phialides size (μm)	Conidia (μm)	Other key characteristics	References
*Cordyceps albocitrinus*	3.8–10.4 × 1.1–1.4 (7.1 × 1.2)		3.3–11.3 × 0.9–1.2 (7.3 × 1.1)	In chains 1–5, ellipsoidal or cylindrical, 0.7–3.5 × 0.6–1.8 (2.1 × 1.2)		Duan, 2019
*Cordyceps amoene-rosea*	90–150 × 2.0–2.5	Verticillate with whorls of 2 to 4	4.0–7.5 × 1.5–3.0, basal portion globose, neck 0.3–0.5	Subglobose to ellipsoidal, irregularly cylindrical, 2.5–3.5 × 1.7–2.2	Synnemata up to 8–10 × 0.3–0.8 mm	Samson, 1974
*Cordyceps araneae*		Solitary or verticillate with whorls of 2–3	5–8 × 1.5–2, basal portion swollen tapering into the apex	Fusoid to ovoid, 3–5 × 1–2		Mongkolsamrit et al., 2020
*Cordyceps bifusispora*		Solitary or verticillate	9–50 × 1.5–2, flask shaped	Globose, ovoid or cylindrical, 1- to 8-celled, 2.5–35 × 2.4–4.5		Liang et al., 2007
*Cordyceps blackwelliae*		Verticillate with whorls of 2–5	(6–)6.5–8(–9) × (2–)2.5–3.5(–4), basal portion globose, neck 2–3 × 1	Cylindrical to ellipsoidal or reniform, (3–)5–7(–8) × 2–3.5	Synnemata numerous, up to 1.5 × 0.5 mm, powdery	Mongkolsamrit et al., 2018
*Cordyceps brevistroma*		Solitary or verticillate with whorls of 2–3	6–9.5(12) × 1.5–2, basal portion swollen or cylindrical tapering into the apex	Ovoid to fusiform, 3–4 × 1–2		Mongkolsamrit et al., 2020
***Cordyceps bullispora***		**Solitary**	**5.6–20.7 × 1.8–3.3**	**Cylindrical or slightly allantoid, oblong-elliptical to ellipsoidal, 4.9–11.1 × 1.9–4.5**	**Asexual morph: *Acremonium*-like and *Mariannaea-*like**	**This study**
*Cordyceps cateniannulata*			3–8 × 1.5–3, basal portion bola, neck 0.5	In chain, ovate to ellipsoidal, 2–3.5 × 1–1.5		Liang, 1981
*Cordyceps cateniobliqua*	90–150 × 1–1.5	Verticillate with whorls of 2–4	8.5–12 × 1 –1.5 and 5–8 × 2–2.5, basal portion ellipsoidal	In chains, long-ovoid and long-ellipsoidal 2.5–7(–12) × 1.5–2	Synnemata unbranched, red	Liang, 1981
*Cordyceps chanhua*		Verticillate with whorls of 2–5	4.2–7 (–13.5) × 2.3–3.5(–5.2)	4.2–7(13.5) × 2.3–3.5(5.2)	Chlamydospores 13–26.5 × 3–12 μm	Li et al., 2021
*Cordyceps chiangdaoensis*			5–22.5 × 1–2	Ovoid to cylindrical, 4–10 × 1.5–2		Tasanathai et al., 2016
*Cordyceps cocoonihabita*	5.8–8.3 × 1.4–2.0	Solitary, alternate or whorled	4.0–16.7 μm long, basal portion 1.5–2.7, neck 0.5–1.2	In chains or solitary, oval to fusiform, 1.6–3.0 × 0.7–1.5		Wang Y. B. et al., 2020
*Cordyceps farinosa*	60–15(300) × 1–1.5		Cylindrical, 7–14 × 1–2	Ovoid or spindle-shaped, 2–2.5(3) × 1–2	Yellow-white powdery spores	Liang et al., 2007
*Cordyceps formosana*	6.0–22.5 × 1.5–2.6	Solitary or verticillate with whorls of 2–3		Ovoid, 2 × 1.5	Hyphae septate, hyaline, 1.9–2.5 μm wide	Olatunji et al., 2018
*Cordyceps fumosorosea*	100 × 1.5–2	Verticillate with whorls of 4–6	5.7–8 × 1–2, basal portion globose or ellipsoidal, neck 0.5	Cylindrical to fusiform, 3–4 × 1–2	Synnemata branched, up to 30 × 0.4 mm, powdery	Liang, 1981
*Cordyceps ghanensis*	90–180 × 2.5–3.5	Verticillate with whorls of 2–4	5.5–8 × 2–3.5, basal portion ellipsoidal, neck, 0.5–0.75	Fusiform, 3.5–5.0 × 2.5–3.0	Synnemata branched, 15–30 × 0.3–0.5 mm, powdery	Liang, 1981
*Cordyceps inthanonensis*		Solitary	(12)14–18.5(20) × 1.5–3, basal portion cylindrical,	Cylindrical, 4–7(9) × 1.5–2		Mongkolsamrit et al., 2020
*Cordyceps jakajanicola*		Verticillate	4–5.3(–6) × 2–3.5(–4), basal portion globose, oval or occasionally conical swollen, neck 0.5	Ellipsoid or cylindrical, (4–)4.5–6(–7) × (1.5–)2–2.5(–3)	Synnemata branched, powdery and floccose	Crous et al., 2019
*Cordyceps javanica*		Verticillate with whorls of 2–5	(6–)9(–10) × 2–2.5, basal portion cylindrical, neck (1–)1.5–3(–4) × 0.5	In chains, fusiform, (3.5–)4(–5) × 2–2.5		Mongkolsamrit et al., 2018
*Cordyceps kuiburienis*		Verticillate with whorls of 2–5	(3–)4–8(–10) × 1.5–2, basal portion swollen, ellipsoidal, necks 1–3 × 1	Ellipsoidal, fusiform, 3–4 × 1.5 –2	Asexual morph: *Evlachovaea*-like	Crous et al., 2019
*Cordyceps lepidopterorum*		Verticillate with whorls of 2–3	5–5.5–8 × 4–5, basal portion globose to flask shaped, neck 2–3 × 1	In chains, ellipsoidal or slipper-shaped, (6–)8–9.5(–10) × 3–4		Mongkolsamrit et al., 2018
*Cordyceps longiphialis*		Solitary	7.0–70.8 × 0.9–2.1	Cylindrical, 2.1–6.0 × 0.8–2.5	Apical conidia more prominent than other conidia, 4.6–10.0 × 1.4–2.3 μm	**This study**
*Cordyceps militaris*		Solitary or verticillate	*Paecilomyces*-type: cylindrical, (0.5–)0.8–1.5 × 6–15(–20), *Verticillium*-type: 0.8–1.2 × (8–)14–20(–25)	Subglobose, 1.5–2 μm, ellipsoidal, 1–2 × 1.5–3		Liang et al., 2007
***Cordyceps nabanheensis***	**Solitary, cylindrical, 4.6–11.5 × 1.6–3.2**	**Solitary or verticillate with whorls of 2 to 3**	**5.6–19.1 × 1.3–3.5**	**Elliptical to oblong, 2.1–4.3 × 1.1–2.7**		**This study**
*Cordyceps neopruinosa*		Solitary or verticillate with whorls of 2–4	(8)10–17.5(20) × 1–2, basal portion cylindrical to ellipsoidal	Irregularly ellipsoidal, cylindrical, (3)4.5–8.5(10) × 1–2	Synnemata awl-shaped, 2–10 × 1 mm	Mongkolsamrit et al., 2020
*Cordyceps ninchukispora*		Solitary or verticillate	30–50 × 2.0–3.0	Ellipsoidal, cylindrical, 0–1 septate, 2.5–10 × 1.5–3		Liang et al., 2007
*Cordyceps parvistroma*		Solitary	10 × 0.5	Cylindrical, (5)7–10(11) × 1– 2(2.5)	Mycelium density	Mongkolsamrit et al., 2020
*Cordyceps polyarthra*				Ellipsoid, 2–2.5 × 1–1.5	Synnemata 30 × 1.0–1.5 mm	Catania et al., 2018
*Cordyceps polystromata*	6.1–43.7 × 1.5–2.9 (27.2–2.1)	Solitary or verticillate	6.2–17.2 × 0.9–2.7 (10.5 × 1.9), basal portion cylindrical or globose	Near-spherical or pseudo-oval, 1.5–3.7 × 1.1–2.5 (2.1 × 1.7)		Duan, 2019
*Cordyceps poprawskii*		Verticillate	5.4–5.6 × 2.4–2.6, basal portion cylindrical to bottle-shaped	Cylindrical to fusiform, 3.9 (2.9–4.6) × 1.6 (1.4–2.1)	Synnemata 5 (2.0–7.0) × 6 (4.0–8.0) mm	Cabanillas et al., 2013
*Cordyceps pruinosa*	6.1–21.6 × 0.9–1. 7 (7.6 × 1.2)	Verticillate	3.7–12.5 × 0.9–1.6 (8.1 × 1.2)	Ellipsoidal, cylindrical, 0.6–1.3 × 0.5–1.0 (1.0 × 0.8, apical conidia two-celled, 7.6–16.3 × 2.0 – 2.5 (11.4 × 2.2)		Liang, 1991; Duan, 2019
***Cordyceps pseudotenuipes***	**Cylindrical, 17.9–25.9 × 1.7–2.1**	**Solitary or verticillate with whorls of 2–5**	**6.8–31.8 × 1.2–3.3**	**Ovoid to ellipsoidal, 3.4–6.5 × 2.2–4.0**	**Chlamydospores 9.2–18.5 × 3.4–7.5 μm**	**This study**
*Cordyceps shuifuensis*	Solitary, cylindrical, 5.5–9.2 × 1.6–2.7		Cylindrical or subulate, 4.7–20 × 0.4–2.1	Macroconidia clavate to oblong-ovate, 5.1–11.8 × 1.3–2.4. Microconidia globose to ellipsoidal, 1.8–3.0 × 1.6–2.5	Asexual morph: *Verticillium*-like	Wang Y. B. et al., 2020
***Cordyceps simaoensis***	**Solitary or verticillate, cylindrical, 17.1–25.2 × 1.4–1.6**	**Solitary or verticillate with whorls of 2–3**	**11.7–50.2 × 3.4–4.0**	**In chains, fusiform or oval, 2.0–4.9 × 2.0–3.3**		**This study**
*Cordyceps spegazzinii*			7–15 × 3	4–5 × 2	Asexual morph: *Evlachovae*a-like	Torres et al., 2005
*Cordyceps subtenuipes*	Biverticillate, 3.5–8.6 × 1.5–2.9	Solitary or verticillate with whorls of 3–6	5.3–42.5 × 1.6–3.4, basal portion cylindrical or flask-shaped	Ellipsoidal or fusiform, 1.9–3.4 × 1.7–2.5		Wang Y. B. et al., 2020
*Cordyceps tenuipes*		Verticillate with whorls of 2–4	6.0–6.5 × 2.5, basal portion subglobose	2–7 × 1.2–2.5		Samson, 1974
*Cordyceps yinjiangensis*	Erect	Verticillate with whorls of 2–3	11.6–18.9 × 1.7–2.2	Cylindrical, multiple-septate, (3.1–)5.3–7.2(–16.1) × (1.6–)2.1(–4.4)		Li et al., 2020

*Boldface: data generated in this study.*

### *Cordyceps bullispora* H. Yu, Q. Y. Dong and Z. Y. Zhao, sp. nov. (Figure 2)

**Mycobank:** MB 842328

**FIGURE 2:**
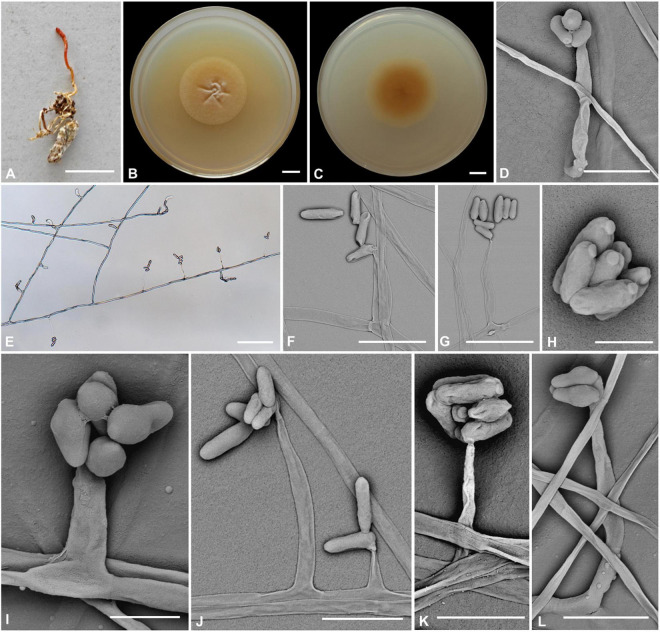
*Cordyceps bullispora*. **(A)** Fungus on the pupa of Lepidoptera. **(B,C)** Colony obverse **(B)** and reverse **(C)** on PDA at 21 days. **(D–G,I–L)** Phialides. **(H)** Conidia. Scale bars: **(A)** = 10 mm; **(B,C)** = 10 mm; **(D)** = 25 μm; **(E)** = 50 μm; **(F,G)** = 15 μm; **(H)** = 3 μm; **(I)** = 5 μm; **(J)** = 15 μm; **(K,L)** = 10 μm.

**Etymology:** Referring to button-like structures on the spores.

**Type:** The Taiji Mountains Nature Reserve, Mizhi Town, Midu County, Yunnan, China. September 20, 2019, H. Yu (YHH 20011, holotype; YFCC 8400, ex-holotype living culture).

**Teleomorph:** Stromata solitary, 10–20 mm long, unbranched, orange-yellow, cylindrical to enlarging apically. The host is covered by a white mycelial surface. Rhizoids flexuous, arising from the head region of host larva buried in soil, 7–10 mm deep under the ground. Stipes cylindrical, white to reddish-orange, 0.1–1.2 mm wide. Fertile parts clavate, orange to reddish-orange, 3.5–7.4 × 0.5–1.5 mm.

**Anamorph:** Two types of conidial arrangement. *Acremonium*-like conidia aggregated in heads at the apex of phialides; *Mariannaea*-like, conidia in imbricate chains, connected laterally.

Colonies on PDA are moderately fast-growing, attaining a diameter of 31–34 mm in 21 days at 25°C, pulvinate, with high mycelial density, Whitish to orange-yellow, reverse deep yellow. Hyphae smooth-walled, branched, septate, hyaline, 1.1–2.7 μm wide. Cultures readily produced phialides and conidia after 2 weeks on potato dextrose agar at room temperature. Phialides arising from aerial hyphae, solitary, 5.6–20.7 × 1.8–3.3 μm, cylindrical, tapering gradually or abruptly toward the apex. Conidia hyaline, one-celled, cylindrical or slightly allantoid, oblong-elliptical to ellipsoidal, 4.9–11.1 × 1.9–4.5 μm.

**Habitat and known distribution:** On larva of Lepidoptera buried below ground at elevation 2000 m in northwestern Yunnan, China.

**Additional specimens examined:** The Taiji Mountains Nature Reserve, Mizhi Town, Midu County, Yunnan, China. On pupae of Lepidoptera, September 20, 2019. YHH 20012, YFCC 8401.

**Comments:** *Cordyceps bullispora* was characterized by unbranched stromata, with cylindrical and orange to reddish-orange fertile parts, Rhizoids flexuous, and the host was the lepidopteran larva. For timing reasons, the fertile part of the specimen was not yet mature at the time of collection in the field. The asexual morph of PDA culture produces cylindrical phialides, which are monothetic, oblong-elliptical to ellipsoidal conidia with a button-like shape.

Based on nr*LSU*, nr*SSU*, *tef-1*α, *rpb1*, and *rpb2* multigene analyses, *C. cocoonihabita* was revealed to have a close relationship with *C. pruinosa* and *C. ninchukispora* (Wang Y. B. et al., 2020). Multigene analyses of ITS, nrLSU, *rpb1*, *rpb2*, and *tef-1*α revealed that *C. neopruinosa* had a close relationship with *C. pruinosa* and *C. ninchukispora* (Mongkolsamrit et al., 2020). *C. cocoonihabita*, *C. neopruinosa, C. pruinosa*, and *C. ninchukispora* all had close relationships, where they shared many similar morphological characteristics, such as they were all characterized by orange- to red-colored stromata and superficial perithecia. *C. bullispora* shared such features, and our phylogenetic analysis indeed indicated that *C. bullispora* was closely relevant to a previously undescribed taxon *C.* cf. *pruinosa* (spat 08-115, spat 09-221) and was separated from *C. cocoonihabita*, *C. neopruinosa. C. pruinosa*, and *C. ninchukispora* in this subclade. However, the perithecia in *C. neopruinosa* were more prolonged and broader than those reported in *Cordyceps pruinosa* and *C. ninchukispora* (330–450 × 150–240 μm vs. 400 × 100 μm vs. 95–145 × 50–60 μm, respectively). *C. cocoonihabita* had a longer stroma. The micromorphological arrangement of conidia was *Isaria*-like characteristics and was significantly different from *C. pruinosa* and *C. ninchukispora*, which had respective morphs of *Mariannaea* G. Arnaud and *Acremonium* Link. *C. bullispora* had rhizoid stromata, superficial perithecia, wider phialides, and longer conidia 4.9–11.1 × 1.9–4.5 μm. The insect host of *C. bullispora* and *C. neopruinosa* all occurred on lepidopteran pupae, and the host of *C. ninchukispora* was the seed of *Beilschmiedia Nees* (Liang, 1983, 1991; Su and Wang, 1986; Mongkolsamrit et al., 2020).

*Cordyceps yinjiangensis* Li et al. (2020) was recently described from Guizhou. Morphologically, it differed from *C. bullispora* by cylindrical and orange to reddish-orange fertile parts, rhizoids several, flexuous, and the host was the lepidopteran larva. The asexual morph from PDA culture produced cylindrical phialides, which were monothetic and oblong-elliptical to ellipsoidal conidia with a button-like character. Tasanathai et al. (2016) reported an anti-pathogenic species, *C. morakotii. C. yinjiangensis* had a close relationship with *C. morakotii* with ant host and conidia formed in an imbricate chain. However, *C. yinjiangensis* was distinct from *C. morakotii*, which had longer phialides (16–20 × 2–3 μm) and bigger aseptate conidia (4–12 × 1–2 μm) (Li et al., 2020).

### *Cordyceps longiphialis* H. Yu, Q. Y. Dong and D. X Tang, sp. nov. (Figure 3)

**MycoBank:** MB 842329

**FIGURE 3:**
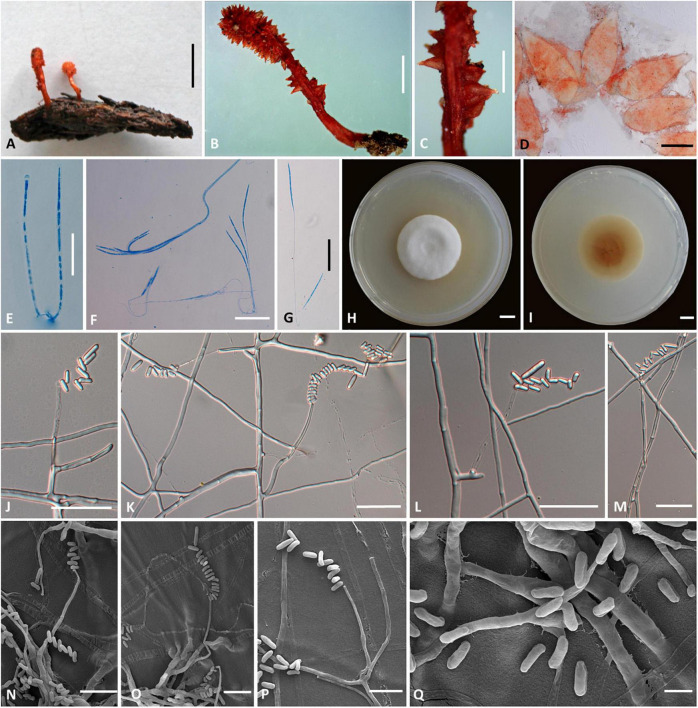
*Cordyceps longiphialis*. **(A,B)** Fungus on the host of Lepidoptera. **(C)** Fertile part. **(D)** Perithecia. **(E)** Asci. **(F,G)** Ascospore. **(H,I)** Colony obverse **(H)** and reverse **(I)** on PDA at 14 days. **(J–P)** Phialide. **(Q)** Conidia. Scale bars: **(A)** = 5 mm; **(B)** = 2 mm; **(C)** = 1 mm; **(D)** = 200 μm; **(E–G,J–M)** = 20 μm; **(H,I)** = 10 mm; **(N–P)** = 10 μm; **(Q)** = 5 μm.

**Etymology:** Referring to its longer phialide than close relationship species in this genus.

**Type:** Xinfang Reservoir, Simao District, Pu’er City, Yunnan, China. September 28, 2020, H. Yu (YHH 20013, holotype; YFCC 8402, ex-holotype living culture).

**Teleomorph:** Stromata (Figures 3A,B) arising from rotten wood, two, unbranched, 13–25 mm long. Stipe cylindrical, 4–10 mm long, 1.5–2 mm in diameter, orange-red to crimson, fleshy, glabrous, smooth. Fertile part single, clavate, covered by a spinous surface, 4–15 mm long, 2.0–2.5 mm in diameter, reddish-orange. Perithecia (Figure 3D) crowded or sparse, crimson-yellowish, superficial, vase-form to oblong, 380–612 × 167.5–268.3 μm. Asci (Figure 3E) 8-spored, fusiform to cylindrical, 113–200 × 1.1–2.7 μm when mature; Ascus caps hemispherical, 1.6–3.6 μm in height, 1.7–3.3 μm in width. Ascospores hyaline, bola-shaped, septate, 110–184 × 0.8–1.3 μm, central region filiform, terminal region narrowly fusiform, do not disarticulate into part-spores.

**Anamorph:** Conidial arrangement *Mariannaea*-like. Colonies on PDA are fast-growing, attaining a diameter of 35–37 mm in 14 days at 25°C, white to pale yellow, cottony, with high mycelial density at the centrum, reverse white to pale yellow. Hyphae smooth-walled, septate, hyaline, 0.8–2.5 μm wide. Cultures readily produced phialides and conidia after 2 weeks on potato dextrose agar at room temperature. Phialides usually solitary on hyphae, basal portion cylindrical to clavate, tapering gradually toward the apex; 7.0–70.8 μm long, 0.6–2.1 μm wide at the base, 0.9–2.1 μm at the middle, and 0.6–1.8 μm wide at the apex. Conidia one-celled, smooth-walled, hyaline, cylindrical, 2.1–6.0 × 0.8–2.5 μm, often formed in an imbricate chain, the size of apical conidia significantly more prominent than other conidia in the chain, 4.6–10.0 × 1.4–2.3 μm.

**Habitat and known distribution:** Buried in rotten logs below ground, in northwestern Yunnan, China.

**Additional specimens examined:** Xinfang Reservoir, Simao District, Pu’er City, Yunnan, China, isolated from stromata of rotten wood at elevation 1,350 m. September 28, 2020, H. Yu (YFCC 8403, ex-holotype living culture).

**Comments:** Phylogenetic analyses showed that *C. longiphialis* was closely related to *C. bullispora*; however, the independent phylogenetic position and different physiological characteristics could distinguish *C. longiphialis* from its sister species, *C. bullispora* (as mentioned above). The distinctive characteristics of *Cordyceps longiphialis* were the cylindrical stromata with a spinous surface, superficial perithecia, much shorter ascospores (110–184 × 0.8–1.3 μm), and much longer phialides (7.0–70.8 μm long). Ascospores of *C. longiphialis* were shorter than *C. neopruinosa* (135–275 × 0.5 μm) and *C. pruinose* (185–200 × 2 μm), but longer than *C. ninchukispora* (90–110 × 1.2 μm). The size of the conidia in *C. longiphialis* was relatively shorter than *C. bullispora*.

### *Cordyceps nabanheensis* H. Yu and Q. Y. Dong, sp. nov. (Figure 4)

**MycoBank:** MB 842341

**FIGURE 4:**
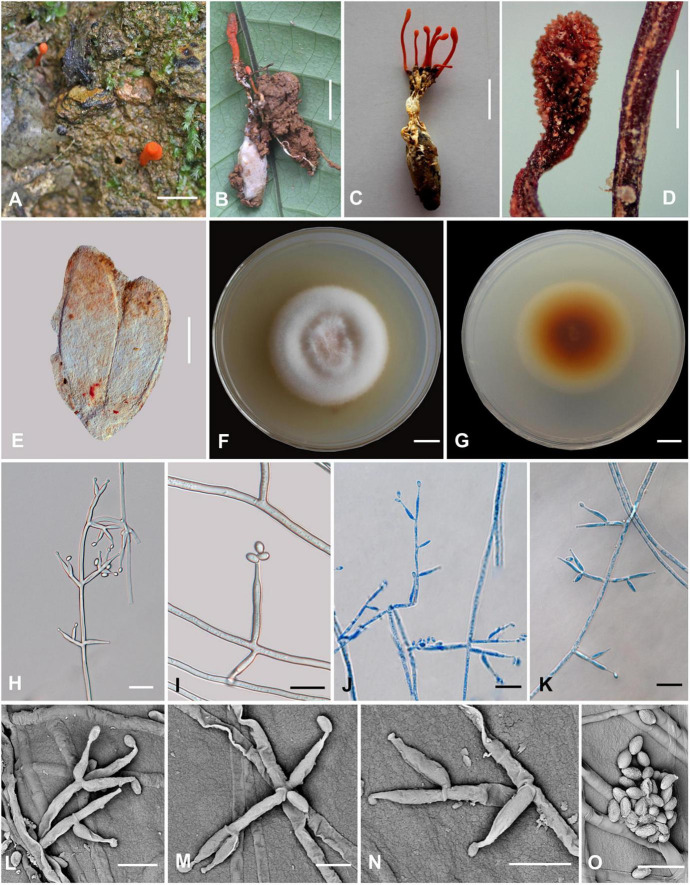
*Cordyceps nabanheensis.* **(A–C)** Fungus on the pupa of Lepidoptera. **(D)** Fertile part. **(E)** Perithecia. **(F,G)** Colony obverse **(F)** and reverse **(G)** on PDA at 14 days. **(I)** Solitary phialide on hyphae. **(H,J–N)** Opposite conidiophore and verticillate phialides. **(O)** Conidia. Scale bars: **(A–C)** = 10 mm; **(D)** = 1 mm; **(E)** = 100 μm; **(F,G)** = 10 mm; **(H,J,K)** = 20 μm; **(I)** = 10 μm; **(L–O)** = 5 μm.

**Etymology:** The location in Nabanhe National Nature Reserve where the species was collected.

**Type:** Manlv village, Nabanhe National Nature Reserve, Jinghong City, Yunnan, China. August 16, 2020, H. Yu (YHH 20019, holotype; YFCC 8409, ex-holotype living culture).

**Teleomorph:** Stromata arising from the pupa of Lepidoptera buried in soil, the host covered by a white mycelial surface, solitary or gregarious, cylindrical to enlarging apically, reddish-orange to crimson, 1.4–2.3 cm long. Rhizoids flexuous, arising from the head region of host larva buried in soil, 7–12 mm deep under the ground. Stipes cylindrical, 12.0–20.9 × 0.6–1.3 mm, fertile parts clavate, 2.0–6.5 × 0.7–1.5 mm. Perithecia superficial, oblong-ovate, 224–322.6 × 71.2–317.3 μm. Asci and ascospores were not observed.

**Anamorph:** Conidial arrangement *Evlachovaea*-like. Colonies on PDA moderately fast-growing, 48–51 mm diameter in 14 days at 25°C, floccose, with high mycelium density; white to orange pinkish, reverse orange-brown. Hyphae smooth-walled, branched, septate, hyaline, septate, 3.5–9.3 μm wide. Cultures readily produced phialides and conidia after 1 week on potato dextrose agar at room temperature showing a powdery appearance due to profuse conidiation. Conidiophores smooth-walled, solitary, cylindrical, 4.6–11.5 × 1.6–3.2 μm. Phialides arising from aerial hyphae, cylindrical or clavate, solitary or in whorls of two to three, tapering abruptly into a narrow neck, 5.6–19.1 × 1.3–3.5 μm. Conidia one-celled, smooth-walled, hyaline, elliptical to oblong, 2.1–4.3 × 1.1–2.7 μm, often formed in an imbricate chain.

**Habitat and known distribution:** On larvae of Lepidoptera buried below ground at elevation 600 m in northeastern Yunnan, China.

**Additional specimens examined:** Manlv village, Nabanhe National Nature Reserve, Jinghong City, Yunnan, China, on larvae of Lepidoptera. August 16, 2020 (YHH 20020, paratype; YFCC 8410 ex-paratype living culture).

**Comments:** *Cordyceps* araneae was firstly reported from Khon Kaen Province, northeastern Thailand by Mongkolsamrit et al. (2020); *C. araneae* was a spider cocoon pathogenetic fungus producing pale orange stromata, perithecia semi-immersed, narrowly ovoid, 450–500 × 150–200 μm with whole bola-shaped ascospores breaking into part-spores 30–65 × 0.5 μm, and developed the *Evlachovaea*-like anamorph, phialides solitary or in whorls of two to three, 5–8 × 1.5–2 μm, conidia fusoid to ovoid, 3–5 × 1–2 μm (Mongkolsamrit et al., 2020).

Based on the ITS, ribosomal large subunit, *rpb1*, *rpb2*, and *tef-1*α genes, multigene analyses revealed that *C. araneae* had a close relationship with *C. kuiburiensis, C. brevistroma*, and *C. nidus*, and they were all characterized by orange to reddish-orange, cylindrical to enlarging apically stromata and a conidial arrangement *Evlachovaea*-like (Chirivi et al., 2017; Crous et al., 2019; Mongkolsamrit et al., 2020). Interestingly, *C. nabanheensis* shared such features, and our phylogenetic analysis indeed indicated that *C. nabanheensis* had a close relationship to *C. araneae* and *C. brevistroma*. However, *C. nabanheensis* and *C. brevistroma* differed from *C. araneae* and *C. kuiburiensis* regarding their hosts. Both *C. nabanheensis* and *C. brevistroma* occur on Lepidoptera larvae, whereas both *C. araneae* and *C. kuiburiensis* occurred on spiders. *C. brevistroma* had bola-shaped whole ascospores, which was the same shape but shorter than *C. araneae* reported (150–200 μm vs. 250–400 μm). In the natural specimen, *C. kuiburiensis* developed the anamorph.

### *Cordyceps pseudotenuipes* H. Yu, Q. Y. Dong, and Y. Wang, sp. nov. (Figure 5)

**Mycobank:** MB 842330

**FIGURE 5:**
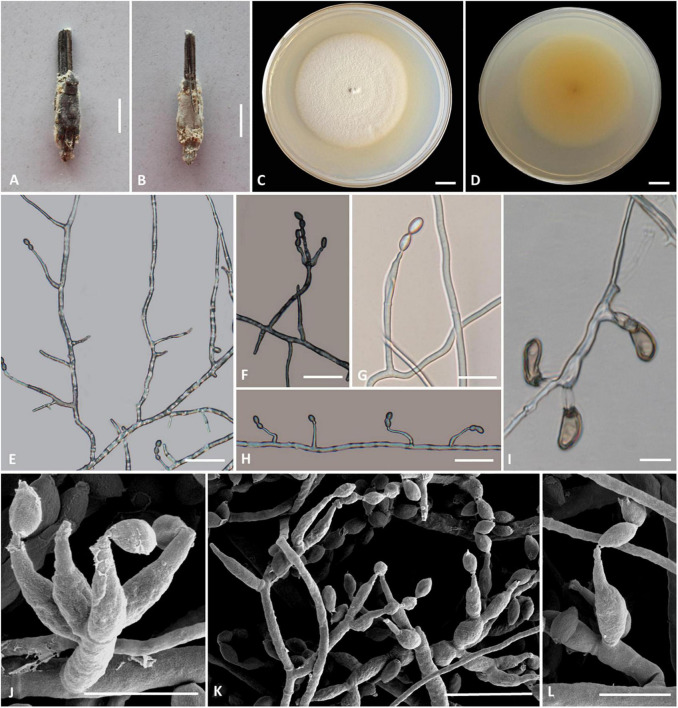
*Cordyceps pseudotenuipes.* **(A,B)** Fungus on the host of Coleopteran. **(C,D)** Colony obverse **(C)** and reverse **(D)** on PDA at 14 days. **(E,G,H,L)** Solitary phialides on hyphae. **(F,J,K)** Conidiophore and verticillate phialides. **(I)** Chlamydospore. Scale bars: **(C,D)** = 5 mm; **(E,F,G)** = 25 μm, **(H,I)** = 20 μm; **(J,L)** = 5 μm; **(K)** = 10 μm.

**Etymology:** Referring to morphological resemblance of *Cordyceps tenuipes* and *Cordyceps subtenuipes* but phylogenetically distinct, “not” *C. tenuipes.*

**Type:** Wild Duck Lake Forest Park, Shuanglong Town, Panlong District, Kunming City, Yunnan, China. September 10, 2019, H. Yu and Y. Wang (YHH 20014, holotype; YFCC 8404, ex-holotype living culture).

**Teleomorph:** Undetermined.

**Anamorph:** Conidial arrangement *Isaria*-like. Synnemata arising from the pupae of Lepidoptera buried in soil, synnemata erect, solitary, flexuous, white, up to 0.5 cm long. Stipes cylindrical, 0.5 mm wide, producing a mass of conidia at the branches of synnemata, powdery.

Colonies on PDA attaining a diameter of 53–55 mm in 14 days at 25°C, white to cream-colored, soft cottony aerial mycelium, reverse pale yellow. Hyphae smooth-walled, branched, septate, hyaline. Synnemata arising from the entire body of larvae were irregularly branched, 0.2–1.0 cm long, 0.1–0.3 mm wide; cylindrical or clavate stipes with powdery white heads. Cultures readily produced phialides and conidia after 2 weeks on potato dextrose agar at room temperature showing a granular appearance due to profuse conidiation. Conidiophores cylindrical, hyaline, smooth-walled, 17.9–25.9 × 1.7–2.1 μm. Phialides from aerial mycelium straight to slightly flexuose, solitary or in whorls of two to five on each branch, cylindrical, usually with a slightly swollen basal part, tapering into the apex, 6.8–31.8 × 1.2–3.3 μm. Conidia hyaline, ovoid to ellipsoidal, smooth, one-celled, 3.4–6.5 × 2.2–4.0 μm. Chlamydospores present, one-celled, solitary, eggplant shape or oval to pyriform, 9.2–18.5 × 3.4–7.5 μm, hyaline becoming brown, thick and smooth-walled.

**Habitat and known distribution:** On the pupa of Lepidoptera buried in the soil. Kunming City, China.

**Additional specimens examined:** Wild Duck Lake Forest Park, Shuanglong Town, Panlong District, Kunming City, Yunnan, China. September 10, 2019, H. Yu (YHH 20015, paratype; YFCC 8405, ex-paratype living culture).

**Comments:** Phylogenetically, *C. pseudotenuipes* formed a separate subclade from the other species of *Cordyceps* with high credible support (100%). *C. pseudotenuipes* was similar to C. tenuipes (Peck) Kepler et al. (2017) based on its conspicuous synnemata and *Isaria*-like asexual conidiogenous structure forming phialides with a swollen basal portion. It differed from *C. tenuipes* by its unbranched synnemata, white color, phialides with a globose basal part, and smaller ovoid to ellipsoidal wider conidia measuring 3.4–6.5 × 2.2–4.0 μm. *C. tenuipes* had multiple synnemata, more giant cylindrical to botuliform conidia with a size of 2.0–7.0 × 1.2–2.5 μm (Samson, 1974). The sexual morph of *C. tenuipes* was proposed as *C. takaomontana* Yakush and Kumaz, with yellowish stromata and being often concurrent with its asexual morph (Liang et al., 2007). However, the sexual morph of *C. pseudotenuipes* was not found in the field.

### *Cordyceps simaoensis* H. Yu, Q. Y. Dong and Z. Q. Wang, sp. nov. (Figure 6)

**MycoBank:** MB 842331

**FIGURE 6:**
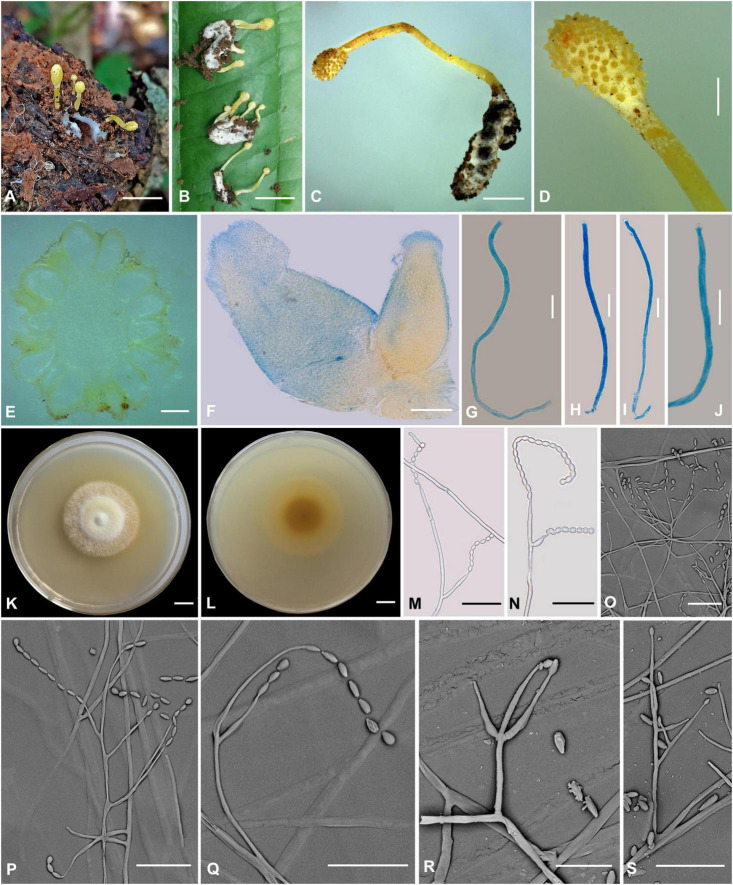
*Cordyceps simaoensis*. **(A–C)** Fungus on the host of Lepidoptera. **(D)** Fertile part. **(E,F)** Perithecia. **(G–J)** Asci. **(K,L)** Colony obverse **(K)** and reverse **(L)** on PDA at 21 days. **(M–S)** Conidiophore and phialide. Scale bars: **(A,B)** = 10 mm; **(C)** = 5 mm; **(D)** = 2 mm; **(E)** = 400 μm; **(F)** = 100 μm; **(G–J)** = 10 μm; **(K,L)** = 10 mm; **(M–S)** = 20 μm.

**Etymology:** The location in Simao District where the species was collected.

**Type:** Xinfang Reservoir, Simao District, Pu’er City, Yunnan, China. September 28, 2020. H. Yu (YHH 20016, holotype; YFCC 8406, ex-holotype living culture).

**Teleomorph:** Stroma arising from the host’s head, solitary or gregarious, mace-shaped, unbranched, 7–25.1 mm in height. Stipe cylindrical, 22 mm long, 1.1–1.2 mm in diameter, bright yellow, fleshy, glabrous, smooth, enlarging abruptly at fertile portion. Fertile portion single, elliptical to fusiform, 1–4.2 mm long, 1.5–3 mm in diameter, bright yellow. Perithecia (Figure 6E) crowded, nearly fully immersed, vase-form, oval to oblong, 638.4–757.6 × 371–531.1 μm, ostioles protruding. Asci (Figures 6G–J) 8-spored, narrowly clavate to nearly cylindrical, 66.9–126.1 × 1.9–2.7 μm; cap 1.2–2.3 μm in height, 2.1–3.5 μm in width. Ascospores not observed.

**Anamorph:** Conidial arrangement *Isaria*-like. Colonies on PDA are fast-growing, attaining a diameter of 40–43 mm at 25°C in 21 days, white to bright yellow, cottony, with high mycelial density at the centrum, forming concentric rings around the inoculum, reverse pale yellow to deep yellow. Hyphae smooth-walled, branched, septate, hyaline, 2.1–2.9 μm wide. Cultures produced phialides and conidia after 45 days on potato dextrose agar at room temperature. Conidiophores are smooth-walled, cylindrical, solitary, or verticillate, 17.1–25.2 × 1.4–1.6 μm. Phialides solitary or verticillate, in whorls of two to three, usually solitary on hyphae, basal portion cylindrical to narrow lageniform, gradually or abruptly tapering toward the apex; 11.7–50.2 μm long, 1.5–3.1 μm wide at the base, 3.4–4.0 μm at the middle, and 0.9–2.0 μm wide at the apex. Conidia is one-celled, hyaline, smooth-walled, fusiform or oval, 2.0–4.9 × 2.0–3.3 μm, often in chains.

**Habitat and known distribution:** On the pupa of Lepidoptera buried in soil.

**Additional specimens examined:** Xinfang Reservoir, Simao District, Pu’er City, Yunnan, China. October 6, 2019, H. Yu (YHH 20017 paratype, YFCC 8407 ex-paratype living culture; YHH 20018 paratype, YFCC 8408 ex-paratype living culture).

**Comments:** Our phylogenetic results demonstrated that the isolated position of this collection was within the *Cordyceps* genus. *C. simaoensis* formed a separate subclade from other species of *Cordyceps* with moderately high credible support (100%). This species was mainly isolated from soil and produced *Isaria*-like asexual morphs and fusiform or oval conidia. *C. simaoensis* was closely related to *C. tenuipes* (Peck) Kepler et al. and might be identified by the size and presence of conidia and its distinct sexual morph. The conidial size of *Cordyceps tenuipes* was 2.0–7.0 × 1.2–2.5 μm, the wider conidia of *Cordyceps pseudotenuipes* was 3.4–6.5 × 2.2–4.0 μm, that of *Cordyceps subtenuipes* was 1.9–3.4 × 1.5–2.7 μm, and that of *Cordyceps simaoensis* was 2.0–4.9 × 2.0–3.3 μm. Cultures of *C. simaoensis* produced phialides and conidia, and it took more days than *C. tenuipes*. Our field observations and herbaria record also indicated that the *C. tenuipes* was widely distributed and significantly ecologically diverse in China, with small bright yellow fleshy stromata as teleomorph and *Isaria*-like anamorph.

## Discussion

Considerable changes to the taxonomy of *Cordyceps* have occurred since the research on entomogenous fungi entered the molecular era. At present, multi-locus phylogenetic analyses have gained importance in delimiting *Cordyceps* species (Tasanathai et al., 2016; Zha et al., 2019; Li et al., 2020, 2021; Wang Y. B. et al., 2020). In this study, most species of the newly circumscribed genus *Cordyceps* were analyzed based on phylogenetic inferences of five nuclear molecular markers (nr*SSU*, nr*LSU*, *tef-1*α, *rpb1*, and *rpb2*). Both ML and BI analyses produced trees with similar topologies that resolved most *Cordyceps* lineages in separate terminal branches. *Cordyceps s*. *s*. was recognized by five statistically well-supported clades, designated as clade I, clade II, clade III, clade IV, and clade V (Figure 1). There were 20 species in clade I. Morphologically, the 20 species shared relatively complicated host, such as spider, Coleoptera, Lepidoptera, Limacodidae, ant, and even plants. They were also complex and varied in shape (ascospores bola-shaped, filiform, ninchukiform, or bifusiform). Clade II was made up of *C. militaris* and other closely related species. With the exception of *C. kyusyuenis* and *C. rosea*, all other species had filiform ascospores; in addition, unlike *C. oncoperae* and *C. rosea*, the rest of the species could easily disarticulate into part-spores. It was found that all the members in clade III had *Isaria*-like anamorphs and all four species in clade IV were described as conidiophores verticillate with phialides in whorls of 2 to 4 or 5, conidia in chains (Table 3). Clade V consisted solely of *Cordyceps grylli*, characterized as pathogenic on Gryllidae adults and relatively larger perithecia (up to 650–810 × 270–370 μm) (Teng, 1963; Liang et al., 2007).

Phylogenetic classifications of cordycepitoid fungi showed that most diagnostic characteristics used in current classifications of *Cordyceps* species (e.g., host, arrangement of perithecia, ascospores fragmentation, conidiogenous structures, conidial shape and size) were not phylogenetically informative (Sung et al., 2007; Kepler et al., 2017; Mongkolsamrit et al., 2018; Wang Y. B. et al., 2020). *Cordyceps lepidopterorum* Mongkolsamrit, Noisripoom, Thanakitpipattana, Spatafora and Luangsa-ard was firstly reported from Chiang Mai Province Thailand by Mongkolsamrit et al. (2018). *Cordyceps chanhua*, which had long been mistaken as *Isaria* (*Paecilomyces*) *cicadae* of a Brazilian specimen, was recently reported as a new species in the genus *Cordyceps s. s.*, for the discovery of its teleomorph in Mt. Jinggang, Jiangxi, China and analyses of both morphological and phylogenetical evidence (Li et al., 2021). Our phylogenetic trees suggested that *C. chanhua* (see Cordyceps cicadae in Figure 1) could not be distinguished from *C. lepidopterorum* (Figure 1). Regarding the morphology, there were no significant differences in the morphological characteristics of anamorph between the two species except for their host (Table 3). Because *C. lepidopterorum* was described earlier than *C. chanhua*, *C. lepidopterorum* should be recommended as the scientific name for this species.

## Data Availability Statement

The datasets presented in this study can be found in online repositories. The names of the repository/repositories and accession number(s) can be found in the article/supplementary material.

## Author Contributions

Q-YD and HY: conceptualization. Q-YD: methodology, writing—original draft preparation, and formal analysis. Q-YD and YW: software and resources.. Z-QW and H-JW: validation. Q-YD, YW, Z-QW, D-XT, Z-YZ, H-JW, and HY: investigation. HY: writing—review and editing and funding acquisition. All authors reviewed and approved the final manuscript.

## Conflict of Interest

The authors declare that the research was conducted in the absence of any commercial or financial relationships that could be construed as a potential conflict of interest.

## Publisher’s Note

All claims expressed in this article are solely those of the authors and do not necessarily represent those of their affiliated organizations, or those of the publisher, the editors and the reviewers. Any product that may be evaluated in this article, or claim that may be made by its manufacturer, is not guaranteed or endorsed by the publisher.
